# Peste Des Petits Ruminants Screening and Diagnostic Tests in African Wildlife in the Context of Rinderpest Eradication (1994–2007)

**DOI:** 10.1155/2023/5542497

**Published:** 2023-06-19

**Authors:** Vladimir Grosbois, Olivier Kwiatek, Nicolas Gaidet, Philippe Chardonnet, Bertrand Chardonnet, Arnaud Bataille, Satya Parida, François Roger, Richard Kock, Geneviève Libeau, Alexandre Caron

**Affiliations:** ^1^ASTRE, University of Montpellier, CIRAD, INRA, Montpellier, France; ^2^SENS, University of Montpellier, CIRAD, Montpellier, France; ^3^International Union for Conservation of Nature, Species Survival Commission, Antelope Specialist Group, Gland, Switzerland; ^4^Consultant, Saint Cloud, France; ^5^Food and Agriculture Organization of the United Nations (FAO), Viale Delle Terme di Caracalla 00153, Rome, Italy; ^6^Department of Pathobiology and Population Sciences, Royal Veterinary College, London AL9 7TA, UK; ^7^Veterinary Faculty, Eduardo Mondlane Universidade, Maputo, Mozambique

## Abstract

Peste des petits ruminants (PPR) virus causes a major disease in domestic and wild small ruminants. Understanding the role of wildlife in PPR virus ecology is important for PPR control and its eradication targeted worldwide in 2030. Developing diagnostic tools that provide reliable data for PPR detection in wildlife will help monitor wild populations for PPR and support the eradication program. We analyze a continental-scale dataset from African free-ranging wild ungulates (*n* = 2570) collected between 1994 and 2007. A Bayesian model estimated the performance of ELISA tests against PPR and rinderpest and their prevalence in African buffalo. The H- and N-ELISA tests used, not initially developed for wildlife, showed poor sensitivities for the detection of PPR antibodies in African buffalo. The estimations of PPR antibody prevalence derived from the results of these tests for animals presumably not exposed or potentially exposed to PPR were uncertain. Thus, poor performances of these PPR serological tests in wildlife would not allow robust estimations of PPR antibody prevalence in African buffalo and would be extremely speculative in non-buffalo wild ungulate species. We recommend that current and new tests be validated for wildlife hosts to provide sufficient sensitivity and specificity of detection and a diagnostic protocol be developed for PPR wildlife research.

## 1. Introduction

Peste des petits ruminants virus (PPRV) belongs to the genus *Morbillivirus* within the *Paramyxoviridae* family closely related to the now eradicated rinderpest (RP) virus [1]. The associated disease (PPR) is one of the most serious and widespread pathologies in domestic small ruminants, representing a major threat for the livelihoods of millions of small-scale farmers across Africa and Asia [2]. Although sheep and goats are primarily affected, it seems that the PPRV can also infect a wide range of wildlife and unconventional hosts [3–5]. However, there has been very limited evidence so far of disease occurrence of PPR in free-ranging wildlife populations in Africa (but see [5, 6]). Evidence of PPR disease occurrence in wildlife is limited to African ungulates in captivity [7] and to severe outbreaks in free-ranging ungulates in Asia [8, 9]. The virus is transmitted mainly through direct contact and survives only briefly in the environment: from 3 to 10 days at 37–40 degrees Celsius up to 3 days in European context; more data are lacking for the African continent [10–12]. The severity of the disease in livestock varies according to the virus strain, the host breed and species and traits (e.g., age and immunity status), and the production system (e.g., intensive, semi-extensive, and free-roaming). Globally, PPR mimics the symptomatology of RP with clinical signs similar to other respiratory syndromes and include coughing, nasal and ocular discharges, and more severe symptoms leading to death in the acute form. It can be assumed that all wild ungulates are susceptible to PPR; however, with regard to the display of clinical signs, only some subfamilies of Bovidae, including Caprinae (only Siberian ibex, *Capra sibirica*) and Antilopinae (currently only two Asian antelopes: saiga (*Saiga tatarica*) and goitered gazelle (*Gazella subgutturosa*)), are known so far to express the disease in free-ranging conditions, in a similar manner to domestic goats and sheep [9, 13] unlike those kept in captivity, for which clinically diseased animals have been reported in a much wider variety of ungulate subfamilies [4, 14, 15]. Camelidae can also express the disease [5]. Convalescent and vaccinated small ruminants develop a strong and lifelong immunity and are protected against re-infection. Cattle show subclinical infection with PPRV with little evidence of viremia and no virus excretion, while they do seroconvert [16, 17].

Food and Agriculture Organization (FAO) of the United Nations and the World Organization for Animal Health (WOAH, founded as OIE) have identified PPR as the next disease to be eradicated worldwide [18–20] with a target of eradication by 2030 [21]. This objective echoes the worldwide eradication of RP in 2011, the first ever eradicated animal disease [22]. Where and when both viruses were cooccurring, ecological interactions between viruses included cross-immunity eliciting cross-reactive antibodies. Thus, in the last stages of the Global Rinderpest Eradication Program (GREP), differential serological diagnosis was required to assess progress towards freedom in both domestic and wild animal populations. For this purpose, different tools were developed and implemented, mainly competitive enzyme-linked immunosorbent assays (ELISAs) [23–25] in association with neutralization assays [26].

PPRV is currently present in the form of four lineages (I–IV) in West, Central, and East Africa, the Middle East, and Asia [1]. The history of its evolution and geographical spread is not completely understood [27] but East Africa has experienced a recent introduction of virus into naive small ruminant populations with high morbidity and mortality (30 to 70%). PPR was first reported in Uganda in 2003 and since 2006–2008, Uganda, Kenya, and Tanzania officially recognized the infection and have been severely impacted by recurrent outbreaks [28–30]. Today, the virus is threatening Southern African countries with Angola, Burundi, and the Democratic Republic of Congo already affected, Mozambique, Rwanda, and Zambia with outbreaks close to international borders however without disease cases, and Malawi, Namibia, and Zimbabwe at a high risk of PPR introduction [31].

Among the potential challenges to successful eradication of PPR worldwide is the understanding of the role of wildlife in PPRV ecology and PPR dynamics [4, 32]. This is particularly important in the socioecological context of rural Africa and Central-South Asia where domestic stock coexists with a large diversity of wild ungulate species, many of them being susceptible to PPR [33]. These contexts of wildlife-livestock interfaces provide ample opportunities for virus sharing between wild and domestic hosts [4, 9, 30, 34–36]. So far, our knowledge about the role of wildlife in PPRV ecology is limited to (i) outbreaks in *ex situ* populations in zoos or fenced enclosures that provide some indication of species susceptibility (e.g., [37, 38]) recently reviewed in Munir [39]; Parida et al. [1]; and Fine et al. [4] and (ii) occasional *in situ* outbreaks in wild mountain Caprinae (Siberian ibex) and in two antelopes (saiga and goitered gazelle) in Mongolia [8, 9, 40].

To better control the disease, improve small ruminant economies, and prevent biodiversity decline, it is vital to identify potential maintenance and bridge hosts among wildlife and to improve epidemiosurveillance methodologies and testing systems for wildlife populations. Failure to do so might also ultimately compromise eradication programs. Although it is likely that the majority of infection cycles are maintained within and between domestic livestock, some wildlife species or communities may be able to maintain PPRV for variable periods of time [41]. Wildlife populations could also act as bridge hosts for PPRV, linking otherwise unconnected infected and naive domestic ungulate populations [42]. Finally, wildlife populations (none of them vaccinated) could be used as sentinel populations for PPRV circulation in regions where vaccination programs are undertaken in domestic stock [43], a strategy that was successfully implemented during the RP eradication program [44].

During the rinderpest eradication campaign conducted across Africa between 1994 and 2007, a large number and diversity of wild ungulates were sampled and tested for both RP and PPR viruses: 2570 serum samples were collected from 48 taxa (species and subspecies). The general objective of the wildlife component of the RP eradication campaign was to clarify the regional RP epidemiological status in Africa, since unequivocal data on wild virus circulation could be obtained from non-vaccinated wildlife sentinels. Because of the cross-reactivity between RP and PPR, samples were tested serologically for antibodies directed against RP antibodies and PPR antibodies. Hence, this continental-scale dataset from African wild ungulates provided an opportunity to explore the role of wildlife in PPR epidemiology since (i) diagnosis on both diseases was advised by international institutions and (ii) wildlife surveillance was used as a tool for RP control.

By using this comprehensive multihost dataset, our goal was to retrospectively estimate the seroprevalence of PPR and RP in African buffalo (*Syncerus caffer*) and other wildlife species across time and in the sub-Saharan African countries. To achieve this objective, the performance in terms of sensitivity and specificity of the four ELISA tests implemented was estimated using a Bayesian model where prior distributions for sensitivity and specificity parameters were specified based on virus neutralization reference tests results obtained from a subset of the collected samples [45]. We discuss the strengths and limitations of the study knowing it was not designed in terms of (i) spatiotemporal sampling strategy (non-probabilistic sampling or empirical sampling) and (ii) targeted wildlife species and adequacy of the screening tests used to answer questions about PPR prevalence. Indeed, wildlife samples were tested with ELISA tests that were validated only for domestic animals.

## 2. Materials and Methods

### 2.1. Data Collection

The African Wildlife Veterinary Project in the Pan African Rinderpest Campaign (PARC) program was initiated by African Union/IBAR, supported by European Union and implemented by the consortium represented by CIRAD (International Centre for Agricultural Research for Development) and ZSL (Zoological Society of London), and this work was subsequently consolidated with the activities of the Epidemiology Unit of the Program for the Control of Epizootic Diseases (PACE) funded by the European Union and other donors. Data collection on wildlife was conducted under the authority of the African Union (AU IBAR) and countries' agreements within this institution. International wildlife experts based in regional AU IBAR offices (in Bamako and Nairobi) carried out field operations in close collaboration with national experts from relevant ministries (mainly agriculture and environment) who facilitated local authorizations and logistics and participated in the data collection.

Wildlife serological data were collected in the frame of the successive PARC and PACE which took place from November 1997 to June 2007. The dataset included samples taken by the Kenya Wildlife Services Veterinary Unit supported by the PARC program during the widespread rinderpest epidemic in Kenya and Tanzania affecting wildlife. Subsequently, more extensive survey work was undertaken in West, Central, and East Africa within the African Wildlife Veterinary Project (1998–2000) and then by the epidemiology unit of the PACE program at AU IBAR based in Nairobi [46]. The objective of this wildlife surveillance was to sample wildlife populations in key ecosystems and assess their historic RP status (Table 1). This was based on (i) ageing the sampled animals, (ii) collecting an age stratified sample in each population, and thereby (iii) assessing from age structured antibody prevalence the date of the latest likely rinderpest virus circulation in that population. Within the overall wildlife dataset (48 species, *n* = 2573, 14 countries), we decided to work on the buffalo subdataset (1 species, *n* = 1211, 10 countries) during the period 1997–2007 because the buffalo sample size was substantial, whereas sample sizes in other species were too small for making proper subdatasets. The number of sampled individuals per country is presented in Table 2.

### 2.2. Testing Methodology

To look for evidence of RP or PPR infections, competitive ELISA (c-ELISA) tests were applied to all serum samples. For both diseases, the H- and the N-ELISA tests were used based on the use of monoclonal antibodies (Mab) targeting either the hemagglutinin protein or the nucleoprotein prepared at Pirbright [24] and at CIRAD [23, 25], respectively. If cross-reactivity occurred with ELISA tests, virus neutralization tests (VNTs) specific either to RP or PPR were implemented. Although cumbersome and not readily adaptable to large-scale surveys, VNT is the reference test for international trade in the WOAH (founded as OIE) Terrestrial Manual [26, 47]. Thus, the status of each sampled animal was derived from the cumulative set of these interpreted results.

In the first step, the following ELISA-based tests were applied on sera: the RP H-ELISA and the PPR H- and N-ELISA. However, along the project, a lack of sensitivity in cattle became a matter of concern with the RP H-ELISA, although highly specific, due to the use of crude virus as an antigen and a Mab raised against the vaccine RBOK strain. The RP N-ELISA is based on the expression of a recombinant N protein as antigen and the related N-based Mab and had been developed earlier in 1992. However, it had been underused because the RP H-ELISA was already commercialized under a kit format. Later on, it was decided to use both RP ELISA tests. For all c-ELISA, the cutoff was settled at 50%. As for differential VNT, threshold was set at 1/10 based on successive 1/2 dilutions of sera from 1/5 to 1/320 (i.e., 1/5, 1/10, 1/20, 1/40, 1/80, 1/160, and 1/320). The test was considered as conclusively positive for one of the two viruses when the neutralization titre for that virus was at least 1/10 and with a difference of at least two levels with the neutralization titre of the heterologous virus. The combination of tests implemented is documented in Table 3. All the samples were subjected to the RP H-ELISA and the PPR N-ELISA tests. Some samples were also tested with RP N-ELISA and/or PPR H-ELISA. Finally, a fraction of the samples was also subjected to the differential VNTs. These samples comprised (i) all the samples which were positive (see below) according to the RP H-ELISA, (ii) most of the samples which were positive (see below) according to the PPR N-ELISA, and (iii) some samples which were negative according to both the RP H-ELISA and the PPR N-ELISA.

### 2.3. Approach to Manage the Dataset

The test result data were stratified according to the applied c-ELISA tests and the PPR and RP status according to the differential VNTs. The number of samples in each category is presented in Table 3. The data were also stratified according to the presumed exposure status of the sampled animal with regard to RP and PPR. The sampling date, the age of the animal at sampling, and the years of the last RP case report and of the first PPR case report in domestic ungulates in the country where the sample had been collected were considered. It was considered that, given the sampling date and its age at this date, an animal should not have been exposed to RP viruses if its estimated birth date was more than two years after the last RP case report in domestic ungulates in the country where the sample had been collected. The 2-year threshold was selected, considered adequate in the absence of virus circulation after the last known outbreak. Conversely, it was considered that, given the sampling date and its age at this date, an animal could have been exposed to RP viruses if it was estimated to be born before the last RP case report in domestic ungulates in the country where the sample had been collected. The same reasoning applies to PPR. The distribution of samples in the different countries covered by the database is presented according to the RP and PPR presumed exposition status in Supplementary Materials S2 and S3, respectively. Ageing of buffalo is accurate to within months up to the age of six years old, based on dentition after which time, ageing becomes dependent on horn shape and other less specific factors [48]. The samples collected less than 3 years before the first PPR case report in domestic ungulates were considered as doubtful regarding potential exposure to PPRV, and the samples from animals born less than 3 years after the last RP case report in domestic ungulates were considered as doubtful regarding potential exposure to RP virus. The 3-year threshold was chosen to take into account the possibility of undetected outbreaks in domestic ruminants and unknown exposure context for wildlife.

### 2.4. Descriptive Analysis

The data were explored before elaborating a statistical model to estimate prevalence and test performance parameters. Firstly, the sample distribution of percent inhibition values of the c-ELISA tests to detect RP and PPR antibodies was plotted for different potential exposure contexts. Secondly, the outcomes of the differential VNTs (when available) were considered through the potential exposure contexts of these samples (see above) and to the results of the differential VNTs available in the same sample cluster (samples collected in the same place on the same date and thus likely to be from the same buffalo population). This procedure would allow detecting incoherent differential VNT results and assess the reliability of this test. Thirdly, the sample distributions of percent inhibition values of the c-ELISA tests for samples with conclusive VNTs were plotted.

### 2.5. Bayesian Model

A subset of the African buffalo serological data was selected to fit the Bayesian model. It included only the samples which had been collected either in a RP potentially exposed and PPR presumably free context or in a RP presumably free and PPR potentially exposed context. This resulted in a reduction in sample size from 1211 to 768. The objective of the analysis was to get estimations of PPR and RP serological prevalence in African buffalo in contrasted contexts regarding potential exposure to the virological agents. The stratification of the data according to the exposure status allowed generating categories among which serological prevalence should differ. This is an important condition in order to be able to assess the performances of serological tests in the absence of gold standard. Moreover, it could allow evaluating whether PPR would circulate in African buffalo before the emergence of the disease in domestic ruminants and whether rinderpest would still circulate in African buffalo after its eradication in domestic ruminants.

The following assumptions were made:

- It was assumed that because of cross-protection between PPR and RP and because the selected samples had been collected in contexts in which only one of the diseases was believed to circulate (see above), no individual can have both antibodies against PPR and against RP. Hence, there were three possible serological statuses: seropositive for PPR and seronegative for RP, seropositive for RP and seronegative for PPR, and seronegative for both PPR and RP. For any potential exposure group, the frequencies of these three possible states summed to 1.
- Because of cross-reactivity, a positive c-ELISA test for one of the diseases can reflect the presence of antibodies against the other disease.
- There is no conditional dependency of results of the different c-ELISA tests.

#### 2.5.1. Likelihood Function

The response variable was a series of binary (negative or positive) c-ELISA test results (RP H-ELISA, RP N-ELISA, PPR H-ELISA, and PPR N-ELISA, referred to as *T*1, *T*2, *T*3, and *T*4). There were thus 2^4^ (*i.e.,* 16) possible outcomes when all the tests had been applied, 2^3^ (*i.e.,* 8) when only three tests had been applied, and 2^2^ (*i.e.,* 4) when only two tests had been applied. The frequencies of these outcomes were considered as realizations of multinomial distributions with 16, 9, or 4 probability parameters.

Each multinomial probability parameter was a function of RP and PPR prevalence (*prev_PPR, prev_RP*) and of sensitivities and specificities of the c-ELISA tests. To account for cross-reactivity, two sensitivity parameters were considered for each c-ELISA test: *Se_PPR* was the probability of a positive test outcome for an individual for which true status was seropositive for PPR while *Se_RP* was the probability of a positive test outcome for an individual for which true status was seropositive for RP. For each test specificity, *Sp* was defined as the probability of a negative test result for an individual for which true status was negative for both PPR and RP. The relationships between the probabilities of the multinomial distribution and these parameters are provided in Supplementary Material S1.

#### 2.5.2. Prior Distributions

Test sensitivities and specificities prior distributions were determined based on the comparison of the c-ELISA test outcomes with the outcomes of the differential virus neutralization test in samples for which this latest test had been conclusive. Concerning estimation of prevalence, for each potential exposure category, a non-informative *Dirichlet* prior distribution (*i.e., Dirichlet* (1,1,1)) was used to account for the constraint that the sum of prevalence should be below 1 (this constraint is the consequence of the cross-immunity assumption which implies that an individual is either positive for RP and negative for PPR, or positive for PPR and negative for RP, or negative for both PPR and RP).

#### 2.5.3. Model Implementation

The model was implemented in *OpenBUGS* [49]. It was fitted to all data strata simultaneously. Three Monte Carlo Markov Chains were simulated using the Metropolis–Hastings algorithm [50]. Chain mixing, unimodality of posterior distributions, and Gelman–Rubin convergence diagnostic were checked to assess model convergence [51].

#### 2.5.4. Determination of *α* and *β* Parameters

**Figure 1:**
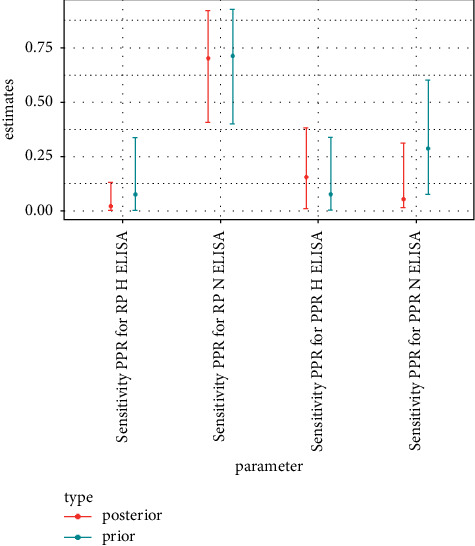
Median, 2.5% quantile, and 97.5% quantile of prior and posterior distribution of sensitivity towards PPR parameters of c-ELISA tests.

**Figure 2:**
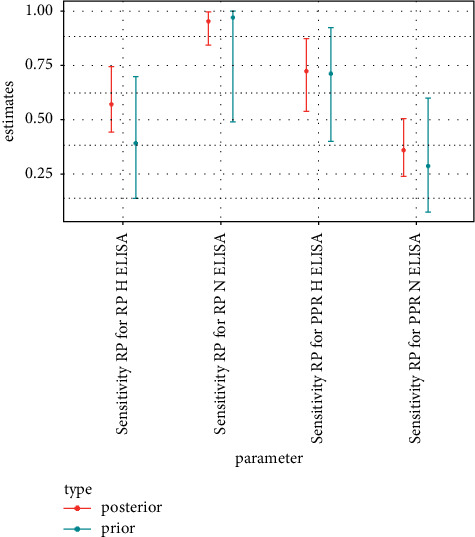
Median, 2.5% quantile, and 97.5% quantile of prior and posterior distribution of sensitivity towards RP parameters of c-ELISA tests.

**Figure 3:**
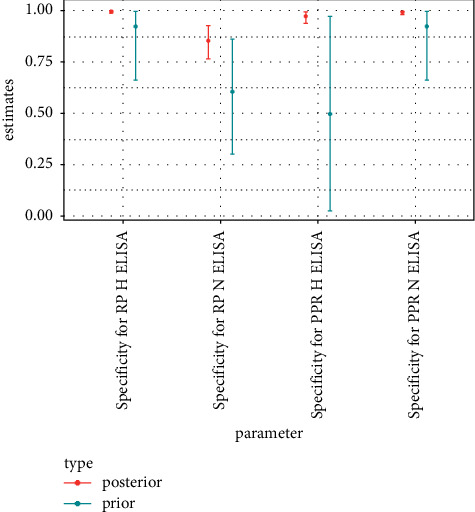
Median, 2.5% quantile, and 97.5% quantile of prior and posterior distribution of specificity parameters of c-ELISA tests.

**Figure 4:**
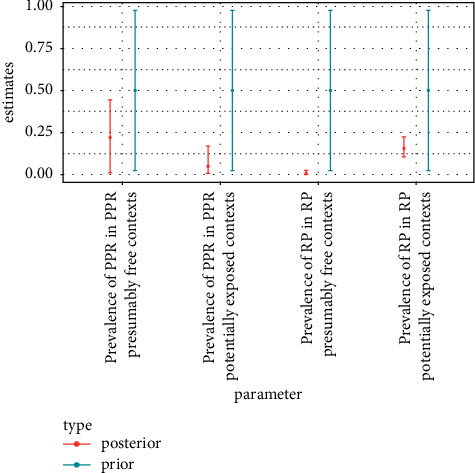
Median, 2.5% quantile, and 97.5% quantile of prior and posterior distribution of PPR and RP serological prevalence parameters.

The *α* and *β* parameters of a beta distribution for probability parameters *p* (here sensitivities and specificities) can be thought of as the number of positive and negative outcomes generated by a binomial process of parameters *p*, *α* *+* *β*. The numbers of positive and negative c-ELISA test results for samples with conclusive VNT outcome were thus used to parameterize the prior distributions of c-ELISA test performance parameters. c-ELISA test outcomes of the samples considered as positive for PPR according to the differential VNT were used to evaluate sensitivity against PPR parameters. c-ELISA test outcomes of the samples considered as positive for RP according to the differential VNT were used to evaluate sensitivity against RP parameters. For specificity parameters, the c-ELISA test outcomes of the samples considered as negative for both RP and PPR according to the differential VNT were used. All *α*, *β* pairs were set so that *α* *+* *β* was never larger than 10. The number of test results in the data ranged between 491 and 743, depending on ELISA test considered (Table 3) which is much larger than the sum of parameters used for the beta prior distributions indicated above. Consequently, prior distributions were not overly informative, as can be confirmed by considering the width of the 95% credible intervals of prior distributions (Figures 1–4).

For sensitivity parameters, *α* was chosen as roughly proportional to the number of positive c-ELISA outcomes and *β* as roughly proportional to the number of negative c-ELISA outcomes. For specificity parameters, *α* was chosen as roughly proportional to the number of negative c-ELISA outcomes and *β* as roughly proportional to the number of positive c-ELISA outcomes.

## 3. Results

### 3.1. Descriptive Analysis

The sampling operations were undertaken in 14 countries within 3 regions of Africa, west, central, and east, including 14 countries and over a 14-year period (1994–2007) (Table 1). Overall, 2570 free-ranging individuals of 48 African wild animal taxa (including species and subspecies) were sampled and tested (Table 2). The data were also stratified according to the presumed exposure context of the sampled animal with regard to RP and PPR (based on WOAH—founded as OIE—reports and publications). Considering the progressive eradication of RP and the presence or emergence of PPR during the study period, the estimated age of African buffalo (*n* = 1211, the most represented species in the sample) was considered in relation to the last known and official RP outbreak (Supplementary Material S2) and their sampling time in relation to the first PPR outbreak in the country (Supplementary Material S3). Sampled individuals were tested using 6 serological tests (virus neutralization test, hereafter referred to as VNT—the gold standard, H- and N-ELISA) for both RP and PPR. However, the 6 tests were not applied systematically to each sample because the objectives of the RP eradication campaign varied during the 14 years of the data collection, and some ELISA tests were either required or not during distinct phases. As a result, the dataset does not provide 6 test results for each sample (Table 3).

All serum tested for the ELISA tests targeting PPRV antibodies were negative for species belonging to the Aepycerotinae (*n* = 58), Antilopinae (*n* = 158), Cephalophinae (*n* = 20) Bovidae subfamilies, and the Giraffidae (*n* = 99), Suidae (*n* = 289) and Felidae (*n* = 6) families, either (i) because no survivor was found after an established infection (however, no PPR outbreak in these subfamilies was ever observed in the wild), (ii) because behaviour, ecology, and size of the population did not create the opportunity for infection, (iii) because of the lack of receptivity to PPR infection, or (iv) because the ELISA tests were not adapted. Positive results for the ELISA tests targeting PPR antibodies have been obtained in this study in species belonging to the Reduncinae (i.e., waterbuck, 1/77), Hippotraginae (roan antelope, 1/39), Alcelaphinae (i.e., Western hartebeest, 1/5: lelwel hartebeest, 1/75; tiang, 1/23), and Bovinae (i.e., bushbuck, 2/22; African buffalo, 56/1211) subfamilies of Bovidae.

**Figure 5:**
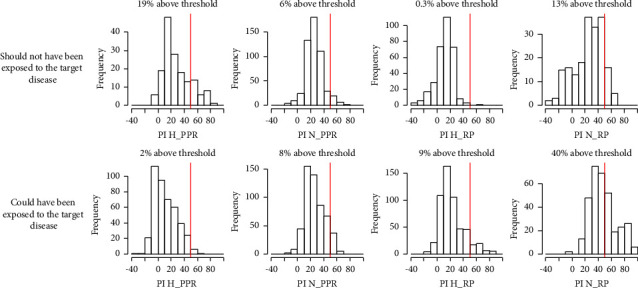
Distribution of percent inhibition values of c-ELISA tests depending on the exposure status of the animals. For PPR, the animals that should not have been exposed to the virus are those that have been sampled at least three years before the first PPR outbreak record in the country while the animals that could have been exposed to the virus are those that have been sampled after the first PPR outbreak record in the country. For RP, the animals that should not have been exposed to the virus are those that are born (based on determination of age at sampling) at least three years after the last RP outbreak record in the country while the animals that could have been exposed to the virus are those that are born before the last RP outbreak record in the country.

The sample distributions of percent inhibition of the 4 c-ELISA tests in the different potential exposure contexts revealed an unexpected pattern for the PPR H-ELISA test and to a lesser extent for the PPR N-ELISA test (Figure 5). Indeed, higher percent inhibition values for the H-ELISA test towards PPR were observed in samples from animals presumed to have not been exposed to PPR (sampled more than two years before the first PPR case report) as compared to samples from animals that could have been exposed to PPR (sampled after the first PPR case report). As for the N-ELISA test towards PPR, the distributions of percent inhibition in the two potential exposure contexts were fairly similar. Sample distributions of percent inhibition for the c-ELISA tests towards RP were more in agreement with the potential exposure status of the samples.

Among the 80 samples which had been subjected to the differential VNTs and for which this test had been conclusive (i.e., to be PPR positive, the PPR titre with cross neutralization was at least two levels above the RP virus titre), 9 presented an outcome of this test that was incompatible with the potential exposure status of the sampled animal or with conclusive differential VNTs obtained in animals from the same cluster. The results of the differential VNTs for these samples were subsequently considered as inconclusive. Furthermore, because the results of the differential VNTs could thus not be considered as fully reliable, this test was not considered as gold standard. Nonetheless, it was considered as reliable enough to be used to specify prior distributions for the c-ELISA tests in the Bayesian model.

**Figure 6:**
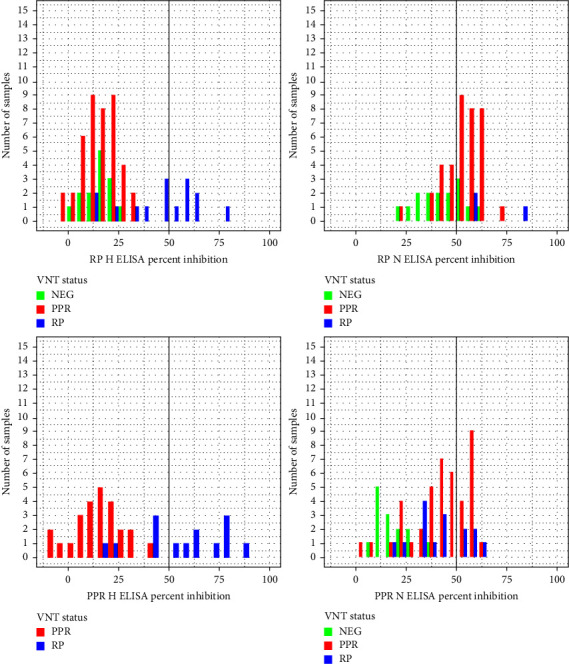
Each of the four panel focuses on the performance of a serological test. For a given serological test, the individuals that were subjected to that test and to the virus neutralization test (VNT) for both PPR and RP are considered. These individuals are classified into three categories according to the outcome of the differential VNT. For each of these categories, the distribution of percent inhibition for the focal serological test is represented. The vertical line represents the cutoff value for percent inhibition above which the individual is considered positive for the target antibodies.

The 71 samples with reliable VNT results (because they were compatible with the potential exposure context of the sampled animal and with the outcomes of the differential VNTs for other animals in the same cluster) were used to plot the distributions of percent inhibition of the c-ELISA tests for samples of different serological status according to the differential VNT results (Figure 6). For the PPR H- and RP H-ELISA, the only samples with percent inhibition >50% (and thus considered positive according to these tests) were samples positive for RP according to the differential VNTs. For the PPR N-ELISA and the RP N-ELISA, the samples with percent inhibition >50% (and thus considered positive according to these tests) included both samples positive for RP and samples positive for PPR according to the VNTs. RP N-ELISA was the only c-ELISA test that produced positive results among samples that were negative for both PPR and RP according to the differential VNTs. These patterns suggest serious deficiencies in the performances of the c-ELISA tests, especially regarding the sensitivity towards PPR of the tests targeting PPR antibodies and regarding cross-reactivity.

The frequency tables of outcomes of the c-ELISA tests (positive or negative) by serological status according to the differential VNTs (positive for PPR or positive for RP or negative) were used to define the prior distributions of the c-ELISA tests' performance parameters (Table 4). The 2.5% and 97.5% quantiles of the resulting prior distribution are plotted along the 95% credible intervals obtained from the posterior distribution in Figures 1–4.

### 3.2. Tests' Performance

Test performance parameter estimations are presented in Table 5 and Figures 1–3. The approach used generated uncertain estimations of sensitivity towards PPR for the two c-ELISA tests targeting PPR antibodies. However, 95% credible intervals for these two tests were below 0.5, reflecting poor sensitivity. By contrast, the estimations of sensitivity towards RP for the two c-ELISA tests targeting RP antibodies were medium and high for the H-ELISA and N-ELISA tests, respectively, with reasonable uncertainty levels. Sensitivity estimations also revealed severe cross-reactivity issues with high estimations for sensitivity towards RP for the H-ELISA test targeting PPR and for sensitivity towards PPR for the N-ELISA test targeting RP as well as medium estimation for sensitivity towards RP for the N-ELISA test targeting PPR. The RP H-ELISA was the only test for which the estimation of sensitivity towards the non-targeted disease's antibodies was low. Specificity (defined in this specific context as the probability of a negative outcome for a sample that is indeed negative for both PPR and RP antibodies) estimations were high for all the c-ELISA tests (although slightly lower for the N-ELISA test targeting RP).

### 3.3. Prevalence Results for African Buffalo

Seroprevalence estimations are presented in Table 6 and Figure 4. Certainly, due to the poor performances of the c-ELISA tests targeting PPR antibodies, the PPR seroprevalence estimation for individuals that should not have been exposed to the PPRV was extremely uncertain. The estimation of PPR seroprevalence for individuals that could have been exposed to the PPRV was less uncertain and low (upper bound of the 95% credible interval at 0.13), suggesting limited circulation of PPRV in buffalo sampled across various populations in West and East Africa.

As expected, the RP seroprevalence estimation for individuals that could have been exposed to the RP virus (born before the last RP outbreak report in the domestic compartment) was larger than the RP seroprevalence estimation for individuals that should not have been exposed to the RP virus (which was close to 0). However, even in the former situation, RP seroprevalence estimation was low (upper bound of the 95% credible interval at 0.24), suggesting limited circulation of RP virus in buffalo sampled across various populations in West and East Africa.

## 4. Discussion

This study analyzes the largest free-ranging wildlife dataset ever explored for PPRV. The results are important considering the current plan to eradicate PPR in Africa, the lack of knowledge about the potential role of African wildlife in PPR epidemiology [4], and the effort and cost it would mean to collect a similar continental dataset. This dataset was however not initially designed to estimate PPR seroprevalence. It also suffers from common biases associated with sampling free-ranging wildlife (e.g., small sample size, diversity of species, sample representativeness of animal populations, and diagnostic tests not developed or adapted for wildlife species) and other biases such as different laboratory standards and quality of the cold chain during transport from the field to the laboratory. However, all tests were performed in reference laboratories and all the wildlife experts involved in data collection—co-authors of this article—have dedicated great efforts to make sure the samples would reach the laboratory in adequate cold chain conditions. We used a Bayesian modelling approach to cope with these issues in assessing the performance of the tests before inferring any epidemiological outcome.

The performances of the sensitivity of c-ELISA tests designed for the detection of PPR in domestic animals and used for wildlife during the RP eradication campaign were poor (Table 5 and Figures 1 and 2): first because their sensitivity towards PPR antibodies was low and second because their sensitivity towards RP antibodies was at the same level or even higher than their sensitivity towards PPR. The application of these tests could thus result in missing many PPR positive individuals and, in contexts where both PPR and RP circulate, in qualifying as PPR positive individuals that are indeed RP positive. c-ELISA tests designed for the detection of RP performed better in terms of sensitivity towards the targeted antibodies (i.e., RP antibodies), especially for the N-ELISA test, but also presented cross-reactivity issues (particularly for N-ELISA test). The only parameter that reflected good performance for all tests (although slightly poorer for the N-ELISA test targeting RP) was specificity defined as the probability of a negative outcome for a sample negative to both PPR and RP antibodies (Table 5 and Figure 3).

As a consequence, the estimation of PPR seroprevalence in wildlife species was only possible for the species with the largest sample size, the African buffalo, and yet with the abovementioned uncertainty, it was impossible to conclude whether or not PPR circulated in African buffalo populations before or after its detection in domestic ungulate populations. Due to the high viral load shared during PPR outbreaks, one might think that PPR ELISAs would perform well during epizootics; however, so far in Africa, PPR spillover from sheep and goats to free-ranging wildlife does not appear to lead to clinical syndromes and much virus expression [30, 36, 52]. This differs from RP where a range of wildlife species expressed disease clinically and viral spread was recorded in their populations. As expected, RP seroprevalence estimations indicate that RP disappeared in African buffalo after eradication of the disease in the domestic compartment. PPR and RP serological test results are provided for a wide range of wildlife taxa (*n* = 48) to inform future research (Table 2). PPR seropositive samples were identified in several taxa belonging to various Bovidae subfamilies, with little data available from suids, perissodactyls, and elephants.

In this study, the c-ELISAs implemented were considered at the time as highly accurate, standardized, and robust, able to measure the immune response due to infection and or vaccination in the respective domestic hosts. These ELISA tests were validated comparatively to VNT, the WOAH (founded as OIE) gold standard test with potentiality to replace it. However, cross-reaction among morbilliviruses is one of the main constraints for achieving a reliable diagnosis. The problem of differential diagnosis is particularly acute with PPR and RP, both viruses overlapping in host range as well as in geographical distribution during RP seromonitoring activities in Africa. Therefore, the PPR and RP VNTs used as a differential test by titrating samples in parallel played a critical role in RP serological surveillance and eradication programs to ensure distinction of the homologous from the heterologous immune response in domestic as well as in wild population [44, 53]. Here, 9 out of 80 VNT tests (11.3%) had to be discarded because of inconsistencies between presence and absence of disease in the area (based on official declarations but with 2-year buffer before first or after last declaration) or between VNT results from other individuals in the same herd (e.g., in Kenya in 2001, 7/8 individuals from the same herd were confirmed RP cases and 1/8 was initially a confirmed PPR case, which is highly unlikely given cross-reactivity between viruses). Those results call for a re-evaluation on the reliability of virus neutralization tests to be used as gold standards.

The estimated low sensitivity of c-ELISA tests for the African buffalo is a matter of concern and questions its use in cattle which are closely related bovines. Despite the relative phylogenetic closeness between wild and domestic ungulates, the ELISA tests did not perform as well with wildlife as in livestock. Our results indicated that the RP H-ELISA was the test with the best sensitivity for PPR (Figure 6 and Table 5). However, since the study took place, the PPR H-ELISA has been removed from the market and the N-ELISA test has evolved. In addition, now that RP is eradicated, cross-reactivity due to this important virus affecting a wide range of ungulates is now excluded. An increase in PPR tests' performance can be expected to improve PPR monitoring in wildlife. Lessening the cross-reactions with other morbilliviruses (e.g., canine distemper virus) in the development of tests designated for the differential serological diagnosis of PPR in domestic as well as in wild population would be highly beneficial. The resulting tools will help to improve our knowledge in the ecology and evolution of PPR viruses and our understanding of the geographical distribution and spread of the disease in specific areas as well as the determinants and drivers of PPR at the interface of populations of domestic and wild animals. Promising methodology for increasing PPR test specificity was developed. They rely on short synthetic peptides representing a single epitope as alternative antigens to recombinant proteins or to the whole microorganism [54–58]. More recently, novel neutralization assays based on pseudotyped heterologous viral species expressing the surface glycoprotein(s) of individual morbilliviruses virus were developed [59]. In this regard, conventional VNT will still be needed to validate new tests to evaluate their diagnostic potential in unexplored populations, camel and different wild species, shown to be susceptible to PPRV [3, 60, 61]. Finally, it needs to be noted that during this study, VNT was only applied when c-ELISAs were positive. This means that there is a bias in the calculation of the relative sensitivity and specificity observed when compiling the results. If VNT had been systematically applied, the level of knowledge of the specificity would have been higher. Indeed, in the dataset, there was no serum which tested negative both for ELISA and VNT. More sera of this kind would increase the specificity ratio.

As for other morbilliviruses (e.g., measles virus (Keeling and Grenfell, 1997) and RP (Rossiter and James, 1989)), the high and long-lasting immunity to PPR infection in recovering animals suggests that large populations are required for maintenance, with sufficient influx of new susceptible hosts, especially young animals. In smaller populations, epidemics may gradually decline until new virus is reintroduced. The high-density domestic populations are therefore considered the most likely source of infection for wildlife [9, 36]. However, some of the regions of Africa sampled in this analysis are characterized by the presence of relatively large wildlife communities (e.g., East African ecosystems such as Greater Serengeti and South Sudan grasslands, where large populations of wild ungulates migrate seasonally) that cohabitated in close proximity with large livestock populations without fencing [6, 30, 36].

Buffaloes were considered as a priority species, including all subspecies, for surveillance of RP. They are phylogenetically close to cattle, they share their susceptibility and sensitivity to RP virus, and they produce antibody detectable by standard cattle serological tests. The large distribution of buffalo was also adequate as a general sentinel and surveillance population for RP in Africa. Similarly, as the related water buffalo in Asia is subclinically infected by PPR and mounts an immune response showing high prevalence of PPR antibodies in endemic situations [64, 65], the African buffalo could also theoretically be used as a sentinel species for PPR. However, the PPR seroprevalence estimations in African buffalo reported here are not very useful in assessing whether this species plays an important role in the maintenance of PPR and should be monitored in PPR surveillance programs. Indeed, the estimations obtained are very imprecise and do not follow the expected pattern as for the comparison of contexts where the sampled animals could vs. should not have been exposed to PPR (Table 6 and Figure 4). The latter challenges the strong assumption that countries would have been free from PPR prior to the first PPR outbreak report in the domestic compartment. However, the estimation of PPR seroprevalence reported here for contexts where African buffalo could have been exposed is very low (95% credible interval [0.001; 0.13], Table 6 and Figure 4). Such low prevalence could result from the fact that many of the buffalo samples were collected in East African areas where, at the time of sampling, PPR had not yet or had only recently been detected in domestic animals. The epidemiological status of buffalo populations regarding PPR could be different now that PPR circulation has been going on for a longer time in the domestic compartment [30, 52]. Moreover, Buffalo may be a dead-end host species for PPRV, as is the case for cattle [30, 36], in which case PPRV transmission within buffalo populations or from buffalo to domestic ungulates would not be possible.

The presence of PPR seropositive healthy animals is an indication of infection and recovery of animals while high prevalence in a herd suggests viral circulation within a species' population. The results presented in Table 2 could suggest a large host species range for PPR in wildlife and inform future PPR surveillance in natural ecosystems notably in species from Alcelaphinae, Reduncinae, and Hippotraginae subfamilies as recently reported [4, 9]. However, sample sizes for these species were too small to estimate sensitivity and specificity of the serological tests in these species so that interpretations of the test results are somewhat speculative and have to be considered with caution. Moreover, future investigations could widen the range of species targeted, including for instance migratory ungulates (e.g., Thomson's gazelle, *Eudorcas thomsonii* in the Serengeti ecosystem, Tanzania and Kenya; Mongalla gazelle, *Eudorcas albonotata* in the Sudd ecosystem, South Sudan).

Many questions remain regarding population/community size thresholds and determinants of PPR maintenance in natural ecosystems comprising a wide range of susceptible hosts, including wild and domestic sympatric populations. Like measles [66] and canine distemper viruses [67], PPR might be maintained through the interaction of multiple interconnected susceptible wild and domestic host communities, acting as one meta-community, each community experiencing intermittent but non-simultaneous PPR epizootics. The socioecological context of wild populations/communities could be a better predictor of its capacity to maintain the virus than its strict species diversity. Furthermore, a range of wildlife species could also link or bridge distant domestic populations and be involved in the spread of PPR across geographical space, without necessarily being able to maintain PPR on the long run [4, 42, 68]. A PPR maintenance model for the domestic compartment has recently been developed and should be adapted to the wildlife compartment and wild/domestic integrated compartments [69]. The RP virus emerged two thousand years ago, the PPR virus two hundred years ago, and the canine distemper virus five hundred years ago in both the terrestrial and marine environments and the measles virus, a human virus, most likely originated from the RP virus. The evolutionary history of morbilliviruses points at plausible emergence of new viruses, given the current host range and geographic coverage of these viruses.

Today, at the beginning of a massive international effort to eradicate PPR globally, the role of African and Asian wildlife in PPR epidemiology is still largely unknown despite recent proofs of ongoing circulation [4]. Considering sheep and goats as the primary hosts for PPRV, the African wild ungulate community is characterized by the near absence of wild sheep and goat species on the continent (except for the walia ibex, *Capra walie,* the Nubian ibex, *Capra nubiana*, and the Barbary sheep, *Ammotragus lervia*, with highly fragmented ranges restricted to isolated mountain massifs), in contrast to the Asian ungulate community, with large populations of numerous taxa spread over immense chains of mountains, a fact that could explain the variability in susceptibility to the disease observed between the continents. Given the results presented here, we recommend (i) longitudinal studies in carefully selected isolated wildlife populations to test the hypothesis of a potential maintenance role of wildlife populations or communities alone and of wildlife populations exposed to wildlife/livestock interfaces (i.e., to explore the maintenance community hypothesis); (ii) virus maintenance modelling in wild and mixed (wild and domestic) host populations to explore host population/community threshold for PPR maintenance; and (iii) the development of a wildlife protocol using new serological tests and re-evaluating the performance of the PPR N-ELISA for wildlife in the current context (i.e., evolution of the test since the study and in the absence of RP). This protocol will need to be validated for key wildlife species (e.g., in Africa, the African buffalo and some selected antelope species such as Grant and Thompson's gazelles). The current candidate tests for this wildlife protocol are pseudotype assays (e.g., [70]), the luciferase immunoprecipitation system (LIPS) [71], a new b-ELISA developed on the African continent [72], and non-invasive PPR diagnostic tests under development for wildlife species [73].

## Figures and Tables

**Table 1:** Sampling occasions per site, time of sampling, and species sampled (African buffalo and other wildlife species) (in the last columns are indicated the last RP and first PPR outbreak officially reported).

	Time of sampling	Region	African buffalo	Other wildlife	Last RP outbreak	First PPR outbreak
Benin	2002-2003	West	17	23	1987	Prior to 1996
Burkina Faso	1999-2000	West	7	33	1988	Prior to 1988
Cameroon	2003	Central	0	2	1986	Prior to 1996
Central Afr. Republic	1999–2004	Central	105	312	1983	1990
Chad	1999–2002; 2004; 2006	Central	89	204	1983	1991
Dem. Rep. of Congo	2002	Central	36	0	1961	2002
Ethiopia	2000-2001	East	22	73	1995	1994
Gabon	2002	Central	4	0	na	1996
Kenya	1994; 1996–2004; 2006	East	559	455	2001	2006
Niger	2003-2004; 2007	West	17	98	1986	1996
Nigeria	2003	West	1	7	1987	Prior to 1975
Sudan	2003-2004	East	3	47	2001	1992
Tanzania	1998–2000	East	197	70	1997	2008
Uganda	1998–2000; 2002; 2004	East	154	38	1994	2003
Total			**1211**	**1362**		

Bold values are totals of the columns.

**Table 2:** Diagnostic results per species: number of animal sampled per species (order/family name are given and tribe for Bovidae family); number of positive (“+”)/number of tested for each of the six diagnostic tests.

Common name	*N*	+	PPR				RP			
Latin name			PPR1	PPR2	PPR3	PPR4	RP1	RP2	RP3	RP4
*Artiodactyla/Suidae*										
Giant forest hog *Hylochoerus meinertzhageni*	1									
Desert warthog *Phacochoerus aethiopicus*	112	**4 RP**	0/2	0/2		0/2			**4/5**	0/2
Common warthog *Phacochoerus africanus*	162	**1 PPR**	**1/3**	0/3	0/3	0/1			0/1	0/1
Red river hog *Potamochoerus porcus*	14				0/1					

*Artiodactyla/Giraffidae*										
Kordofan giraffe *Giraffa camelopardalis antiquorum*	45									
Reticulated giraffe *Giraffa camelopardalis reticulata*	16	**3 RP**							**3/5**	
Masai giraffe *Giraffa camelopardalis tippelskirchi*	28	**5 RP**							**5/15**	

*Artiodactyla/Bovidae*										
(i) *Bovinae/Bovini*										
African savanna buffalo *Syncerus caffer caffer* *S. c. brachyceros* *S. c. aequinoctialis*	1211	**67 PPR** **118 RP**	**14/114**	**13/114**	**12/13**	**28/62**	**13/51**	**24/51**	**80/256**	**1/19**
African forest buffalo *Syncerus caffer nanus*	1									
(ii) ***Bovinae/Tragelaphini***										
Mountain nyala *Tragelaphus buxtoni*	7		0/1	0/1						0/1
Lesser kudu *Tragelaphus imberbis*	17								0/2	
Greater kudu *Tragelaphus strepsiceros*	20								0/3	
Giant eland *Tragelaphus derbianus*	23		0/2	0/2						
Common eland *Tragelaphus oryx*	49	**1 PPR** **9 RP**	0/2	0/2		1/2			**9/13**	0/2
Bongo *Tragelaphus eurycerus*	3									
Bushbuck *Tragelaphus scriptus*	22	**4 PPR** **1 RP**	**1/3**	**1/3**	**2/2**	0/1				**1/1**
(iii) ***Hippotraginae***										
Roan antelope *Hippotragus equinus*	40	**2 PPR**	0/4	**2/4**					0/1	
Sable antelope *Hippotragus niger*	5									
Beisa oryx *Oryx beisa*	14	**2 RP**							**2/2**	
(iv) ***Alcelaphinae***										
Coke's hartebeest *Alcelaphus buselaphus cokii*	18								0/9	
Lelwel hartebeest *Alcelaphus buselaphus lelwel*	75	**3 PPR**	0/10	**2/10**	**1/3**					
Lichtenstein's hartebeest *Alcelaphus buselaphus lichtensteinii*	1									
Western hartebeest *Alcelaphus buselaphus major*	5									
Swayne's hartebeest *Alcelaphus buselaphus swaynei*	11		0/2	0/2		0/1				0/1
Hirola *Beatragus hunteri*	33									
Topi *Damaliscus lunatus jimela*	12	**1 RP**	0/1	0/1		0/1			**1/4**	0/1
Tiang *Damaliscus lunatus tiang*	24	**3 PPR**	**1/2**	0/2	**2/2**					
Blue wildebeest *Connochaetes taurinus*	11									
(v) ***Reduncinae***										
Bohor reedbuck *Redunca redunca*	8									
Waterbuck *Kobus ellipsiprymnus* *K. e. defassa* in West and Central Africa *K. e. ellipsiprymnus* in East Africa	77	**5 PPR**	**1/9**	**4/9**						
Buffon's Kob *Kobus kob kob*	176	**12 PPR**	**1/11**	**5/11**	**6/7**					
White-eared kob *Kobus kob leucotis*	17		0/2	0/2		0/2				0/2
Uganda kob *Kobus kob thomasi*	43									
(vi) ***Aepycerotinae***										
Impala *Aepyceros melampus*	58	**7 RP**							**7/11**	
(vii) ***Antilopinae***									0/0	
Grant gazelle *Nanger granti*	13								0/7	
Dorcas gazelle *Gazella dorcas*	132									
Red-fronted gazelle *Eudorcas rufifrons*	1									
Gerenuk *Litocranius walleri*	1	**1 RP**							**1/1**	
Oribi *Ourebia ourebi*	12		0/1	0/1						
(viii) ***Cephalophinae***										
Blue duiker *Philantomba monticola*	7									
Red-flanked duiker *Cephalophus rufilatus*	6									
Yellow-backed duiker *Cephalophus silvicultor*	2									
Common duiker *Sylvicapra grimmia*	5									

*Artiodactyla/Hippopotamidae*										
Hippopotamus *Hippopotamus amphibius*	1									

*Carnivora/Felidae*										
Lion *Panthera leo*	1		0/1	0/1						
Leopard *Panthera pardus*	5									

*Proboscidea/Elephantidae*										
African elephant *Loxodonta africana*	1									

*Primates/Cercopithecidae*										
Olive baboon *Papio anubis*	2									

The third “+” column represents the number of individuals per species considered positive for each disease according to the following criteria. **PPR1**: positive for PPR according to the differential VNT test in a PPR potentially exposed/RP presumably free context. **PPR2**: positive for PPR according to the PPR VNT test in a PPR potentially exposed/RP presumably free context but difference in titre level with the RP VNT test <2. **PPR3**: positive for PPR according to the PPR VNT test in a PPR potentially exposed/RP presumably free context but the RP VNT test has not been done. **PPR4**: positive for PPR according to the differential VNT test in a PPR potentially exposed and RP potentially exposed or doubtful context. **RP1**: positive for RP according to the differential VNT test in a RP potentially exposed/PPR presumably free context. **RP2**: positive for RP according to the RP VNT test in a RP potentially exposed/PPR presumably free context but difference in titre level with the PPR VNT test <2. **RP3**: positive for RP according to the RP VNT test in a RP potentially exposed/PPR presumably free context but the PPR VNT test has not been done. **RP4**: positive for RP according to the differential VNT test in a RP potentially exposed and PPR potentially exposed or doubtful context. Bold values indicate the number of positive individuals per species for both RP and PPR as international standards for each disease. Bold values in other columns indicate which individuals have been recognised positive and for which test.

**Table 3:** Number of buffalo samples included in the Bayesian model (i.e., including only the samples which had been collected either in a RP potentially exposed and PPR presumably free context or in a RP presumably free and PPR potentially exposed context), indication of ELISA tests performed (a cross indicates that the test referred to in the column has been implemented), and their RP and PPR status in relation to test results (including virus neutralization tests (VNTs)).

ELISA tests				Differential VNT results				
RP H-ELISA	RP N-ELISA	PPR H-ELISA	PPR N-ELISA	Not tested	Negative PPR and RP	Positive for PPR	Positive for RP	Total
x	x	x	x	430	0	24	2	456
x		x	x	185	0	6	11	202
x	x		x	15	10	6	4	35
x			x	49	0	1	0	50
Total				679	10	37	17	743

**Table 4:** Outcomes (number of positive/number of negative) of the c-ELISA tests for samples with conclusive differential virus neutralization test (VNT) outcomes and parameters of the beta distributions used for the prior distributions of the performance parameters of these c-ELISA tests.

c-ELISA test	Outcomes of the c-ELISA test (positives/negatives) depending on the outcome of the differential VNT			Parameters (*α*, *β*) of the priori beta distribution for the c-ELISA test performance parameters		
	VNT PPR positive	VNT RP positive	VNT negative	Se PPR	Se RP	Sp
RP H-ELISA	0/42	7/8	0/14	1/9	4/6	9/1
RP N-ELISA	27/10	3/0	6/8	7/3	3/0.3	6/4
PPR H-ELISA	0/25	9/5	0/0	1/9	7/3	1/1
PPR N-ELISA	15/27	5/10	0/14	3/7	3/7	9/1

**Table 5:** Point estimation (median of the posterior distribution) and 95% credible interval for c-ELISA test performance parameters.

	Se_RP	Se_PPR	Sp
H-ELISA RP	0.58 [0.45; 0.76]	0.02 [0.0007; 0.14]	0.998 [0.991; 0.999]
N-ELISA RP	0.96 [0.85; 0.998]	0.70 [0.41; 0.92]	0.85 [0.78; 0.92]
H-ELISA PPR	0.72 [0.54; 0.87]	0.20 [0.02; 0.43]	0.97 [0.94; 0.99]
N-ELISA PPR	0.37 [0.24; 0.51]	0.05 [0.01; 0.27]	0.992 [0.98; 0.998]

“Se”: sensitivity and “Sp”: specificity.

**Table 6:** Point estimation (median of the posterior distribution) and 95% credible interval for prevalence parameters.

	Prev RP	Prev PPR
RP exposed/PPR free	0.16 [0.11; 0.23]	0.24 [0.02; 0.52]
PPR exposed/RP free	0.007 [0.0005; 0.02]	0.03 [0.001; 0.13]

## Data Availability

The datasets generated and analyzed during the current study are available from the corresponding author on reasonable request. The Bayesian model is available in supplementary information.

## Supplementary Materials

Supplementary Materials Supplementary Material 1: Bayesian model code. Supplementary Material 2: distribution of number of sampled buffalo (*n* = 1211) with regard to their birth year, the country where they were sampled, and the year of the last RP outbreak record. It was considered that, given the sampling date and its age at this date, an animal should not have been “exposed” to RP viruses if its estimated birth date was more than two years after the last RP case report in domestic ungulates in the country where the sample had been collected. When within the 2-year period, the exposure was considered “doubtful.” Vertical bars indicate the year of the last reported RP outbreak in each country. Dashed vertical bars mean that the last RP outbreak report occurred before the indicated year. Supplementary Material 3: distribution of number of sampled buffalo (*n* = 1211) with regard to the sampling year, the country where they were sampled, and the year of the first PPR outbreak record. It was considered that, given the sampling date, an animal could have been exposed to PPR virus if it was estimated to be born after the first PPR case report in domestic ungulates in the country where the sample had been collected. If its sampling date was less than two years before first PPR case report in the country, the exposure context was considered “doubtful.” Vertical bars indicate the year of the first PPR outbreak in each country. Dashed vertical bars mean that the first PPR case report occurred before the indicated year.
