# Supplementary material for: Peste Des Petits Ruminants Screening and Diagnostic Tests in African Wildlife in the Context of Rinderpest Eradication (1994–2007)
Source: Transbound Emerg Dis. 2023 Jun 19;2023:5542497. doi: 10.1155/2023/5542497 (PMC12017071; doi:10.1155/2023/5542497)
Supplement: Supplementary Materials — Supplementary Material 1: Bayesian model code. Supplementary Material 2: distribution of number of sampled buffalo (n = 1211) with regard to their birth year, the country where they were sampled, and the year of the last RP outbreak record. It was considered that, given the sampling date and its age at this date, an animal should not have been “exposed” to RP viruses if its estimated birth date was more than two years after the last RP case report in domestic ungulates in the country where the sample had been collected. When within the 2-year period, the exposure was considered “doubtful.” Vertical bars indicate the year of the last reported RP outbreak in each country. Dashed vertical bars mean that the last RP outbreak report occurred before the indicated year. Supplementary Material 3: distribution of number of sampled buffalo (n = 1211) with regard to the sampling year, the country where they were sampled, and the year of the first PPR outbreak record. It was considered that, given the sampling date, an animal could have been exposed to PPR virus if it was estimated to be born after the first PPR case report in domestic ungulates in the country where the sample had been collected. If its sampling date was less than two years before first PPR case report in the country, the exposure context was considered “doubtful.” Vertical bars indicate the year of the first PPR outbreak in each country. Dashed vertical bars mean that the first PPR case report occurred before the indicated year. [file 5542497.f1.docx]

**Supplementary material**

**Supp. Mat. 1 :** Bayesian model code

+ Prev_PPR*(1-Se_PPR_T1)*Se_PPR_T2*(1-Se_PPR_T3)*(1-Se_PPR_T4)

+ (1-Prev_PPR-Prev_RP)*Sp_T1*(1-Sp_T2)*Sp_T3*Sp_T4

p(-+-+) = Prev_RP*(1-Se_RP_T1)*Se_RP_T2*(1-Se_RP_T3)*Se_RP_T4

+ Prev_PPR*(1-Se_PPR_T1)*Se_PPR_T2*(1-Se_PPR_T3)*Se_PPR_T4

+ (1-Prev_PPR-Prev_RP)*Sp_T1*(1-Sp_T2)*Sp_T3*(1-Sp_T4)

p(-++-) = Prev_RP*(1-Se_RP_T1)*Se_RP_T2*Se_RP_T3*(1-Se_RP_T4)

+ Prev_PPR*(1-Se_PPR_T1)*Se_PPR_T2*Se_PPR_T3*(1-Se_PPR_T4)

+ (1-Prev_PPR-Prev_RP)*Sp_T1*(1-Sp_T2)*(1-Sp_T3)*Sp_T4

p(-+++) = Prev_RP*(1-Se_RP_T1)*Se_RP_T2*Se_RP_T3*Se_RP_T4

+ Prev_PPR*(1-Se_PPR_T1)*Se_PPR_T2*Se_PPR_T3*Se_PPR_T4

+ (1-Prev_PPR-Prev_RP)*Sp_T1*(1-Sp_T2)*(1-Sp_T3)*(1-Sp_T4)

p(+---) = Prev_RP*Se_RP_T1*(1-Se_RP_T2)*(1-Se_RP_T3)*(1-Se_RP_T4)

+ Prev_PPR*Se_PPR_T1*(1-Se_PPR_T2)*(1-Se_PPR_T3)*(1-Se_PPR_T4)

+ (1-Prev_PPR-Prev_RP)*(1-Sp_T1)*Sp_T2*Sp_T3*Sp_T4

p(+--+) = Prev_RP*Se_RP_T1*(1-Se_RP_T2)*(1-Se_RP_T3)*Se_RP_T4

+ Prev_PPR*Se_PPR_T1*(1-Se_PPR_T2)*(1-Se_PPR_T3)*Se_PPR_T4

+ (1-Prev_PPR-Prev_RP)*(1-Sp_T1)*Sp_T2*Sp_T3*(1-Sp_T4)

p(+-+-) = Prev_RP*Se_RP_T1*(1-Se_RP_T2)*Se_RP_T3*(1-Se_RP_T4)

+ Prev_PPR*Se_PPR_T1*(1-Se_PPR_T2)*Se_PPR_T3*(1-Se_PPR_T4)

+ (1-Prev_PPR-Prev_RP)*(1-Sp_T1)*Sp_T2*(1-Sp_T3)*Sp_T4

p(+-++) = Prev_RP*Se_RP_T1*(1-Se_RP_T2)*Se_RP_T3*Se_RP_T4

+ Prev_PPR*Se_PPR_T1*(1-Se_PPR_T2)*Se_PPR_T3*Se_PPR_T4

+ (1-Prev_PPR-Prev_RP)*(1-Sp_T1)*Sp_T2*(1-Sp_T3)*(1-Sp_T4)

p(++--) = Prev_RP*Se_RP_T1*Se_RP_T2*(1-Se_RP_T3)*(1-Se_RP_T4)

+ Prev_PPR*Se_PPR_T1*Se_PPR_T2*(1-Se_PPR_T3)*(1-Se_PPR_T4)

+ (1-Prev_PPR-Prev_RP)*(1-Sp_T1)*(1-Sp_T2)*Sp_T3*Sp_T4

p(++-+) = Prev_RP*Se_RP_T1*Se_RP_T2*(1-Se_RP_T3)*Se_RP_T4

+ Prev_PPR*Se_PPR_T1*Se_PPR_T2*(1-Se_PPR_T3)*Se_PPR_T4

+ (1-Prev_PPR-Prev_RP)*(1-Sp_T1)*(1-Sp_T2)*Sp_T3*(1-Sp_T4)

p(+++-) = Prev_RP*Se_RP_T1*Se_RP_T2*Se_RP_T3*(1-Se_RP_T4)

+ Prev_PPR*Se_PPR_T1*Se_PPR_T2*Se_PPR_T3*(1-Se_PPR_T4)

+ (1-Prev_PPR-Prev_RP)*(1-Sp_T1)*(1-Sp_T2)*(1-Sp_T3)*Sp_T4

p(++++) = Prev_RP*Se_RP_T1*Se_RP_T2*Se_RP_T3*Se_RP_T4

+ Prev_PPR*Se_PPR_T1*Se_PPR_T2*Se_PPR_T3*Se_PPR_T4

+ (1-Prev_PPR-Prev_RP)*(1-Sp_T1)*(1-Sp_T2)*(1-Sp_T3)*(1-Sp_T4)

These equations were simplified for cases where only three or two tests had been applied by removing the terms corresponding to the tests that had not been applied. For instance in the case where the N c_elisa test for RP (*i.e.* T2) had not been applied, the equation for the probability of getting positive results for the three other tests was:

p(+++) = Prev_RP*Se_RP_T1 *Se_RP_T3*Se_RP_T4

+ Prev_PPR*Se_PPR_T1* Se_PPR_T3*Se_PPR_T4

+ (1-Prev_PPR-Prev_RP)*(1-Sp_T1)*(1-Sp_T3)*(1-Sp_T4)

**Supp. Mat. 2**: Distribution of number of sampled buffalo (n= 1211) with regard to their birth year, the country where they were sampled and the year of the last RP outbreak record. It was considered that, given the sampling date and its age at this date, an animal should not have been “exposed” to RP viruses if its estimated birth date was more than two years after the last RP case report in domestic ungulates in the country where the sample had been collected. When within the 2-years period, the exposure was considered “doubtful”. Vertical bars indicate the year of the last reported RP outbreak in each country. Dashed vertical bars mean that the last RP outbreak report occurred before the indicated year.


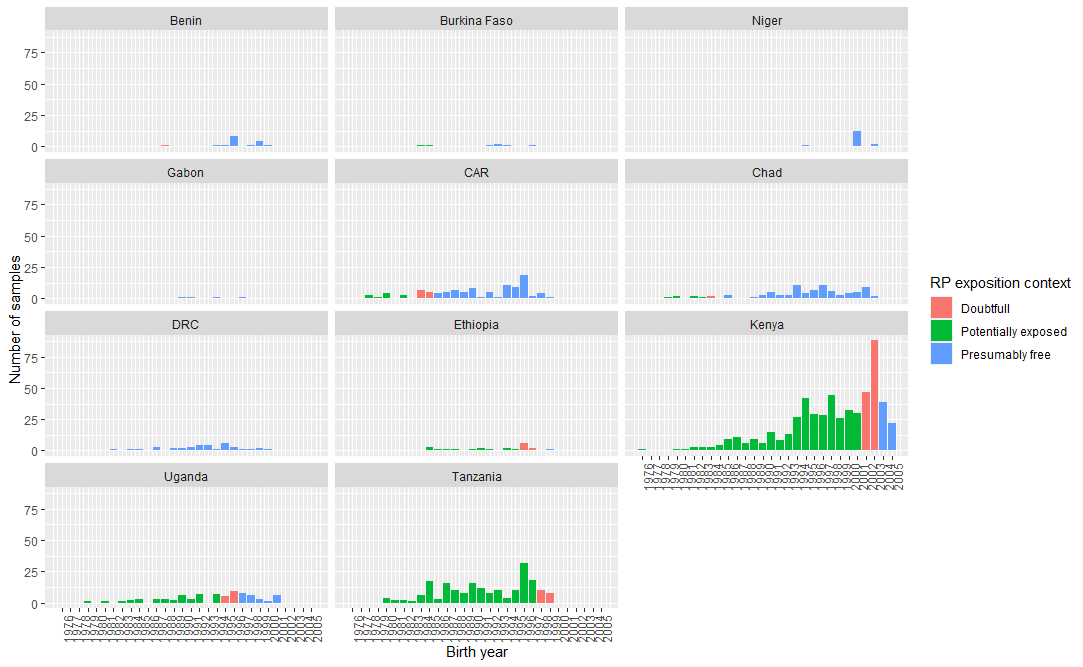


**Supp. Mat. 3**: Distribution of number of sampled buffalo (n=1211) with regard to the sampling year, the country where they were sampled and the year of the first PPR outbreak record. It was considered that, given the sampling date, an animal could have been exposed to PPR virus if it was estimated to be born after the first PPR case report in domestic ungulates in the country where the sample had been collected. If its sampling date was less than two years before first PPR case report in the country, the exposure context was considered “doubtful”. Vertical bars indicate the year of the first PPR outbreak in each country. Dashed vertical bars mean that the first PPR case report occurred before the indicated year.


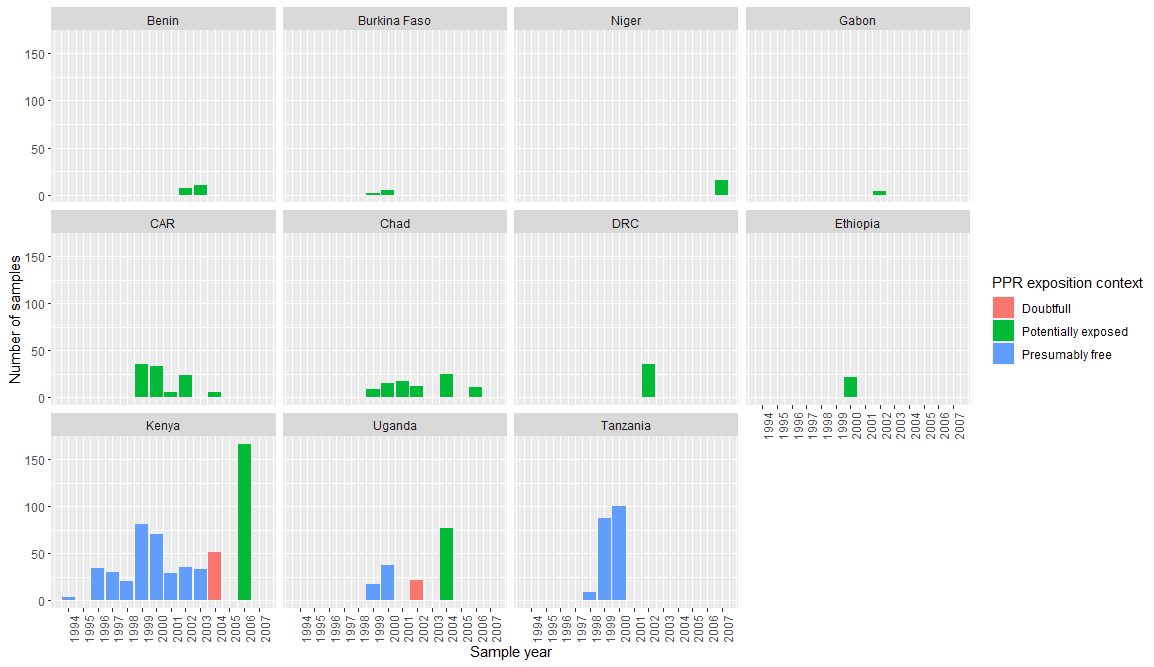


**Supp mAt S1:** The relationships between the probabilities of the multinomial probability law and the parameters of the bayesian model.

p(----) = Prev_RP*(1-Se_RP_T1)*(1-Se_RP_T2)*(1-Se_RP_T3)*(1-Se_RP_T4)

+ Prev_PPR*(1-Se_PPR_T1)*(1-Se_PPR_T2)*(1-Se_PPR_T3)*(1-Se_PPR_T4)

+ (1-Prev_PPR-Prev_RP)*Sp_T1*Sp_T2*Sp_T3*Sp_T4

p(---+) = Prev_RP*(1-Se_RP_T1)*(1-Se_RP_T2)*(1-Se_RP_T3)*Se_RP_T4

+ Prev_PPR*(1-Se_PPR_T1)*(1-Se_PPR_T2)*(1-Se_PPR_T3)*Se_PPR_T4

+ (1-Prev_PPR-Prev_RP)*Sp_T1*Sp_T2*Sp_T3*(1-Sp_T4)

p(--+-) = Prev_RP*(1-Se_RP_T1)*(1-Se_RP_T2)*Se_RP_T3*(1-Se_RP_T4)

+ Prev_PPR*(1-Se_PPR_T1)*(1-Se_PPR_T2)*Se_PPR_T3*(1-Se_PPR_T4)

+ (1-Prev_PPR-Prev_RP)*Sp_T1*Sp_T2*(1-Sp_T3)*Sp_T4

p(--++) = Prev_RP*(1-Se_RP_T1)*(1-Se_RP_T2)*Se_RP_T3*Se_RP_T4

+ Prev_PPR*(1-Se_PPR_T1)*(1-Se_PPR_T2)*Se_PPR_T3*Se_PPR_T4

+ (1-Prev_PPR-Prev_RP)*Sp_T1*Sp_T2*(1-Sp_T3)*(1-Sp_T4)
